# Research on tire crack detection using image deep learning method

**DOI:** 10.1038/s41598-023-35227-z

**Published:** 2023-05-17

**Authors:** Shih-Lin Lin

**Affiliations:** Graduate Institute of Vehicle Engineering, National Changhua University of Education, No.1, Jin-De Road, Changhua City, 50007 Taiwan

**Keywords:** Energy science and technology, Engineering, Mathematics and computing

## Abstract

Driving can understand the importance of tire tread depth and air pressure, but most people are unaware of the safety risks of tire oxidation. Drivers must maintain vehicle tire quality to ensure performance, efficiency, and safety. In this study, a deep learning tire defect detection method was designed. This paper improves the traditional ShuffleNet and proposes an improved ShuffleNet method for tire image detection. The research results are compared with the five methods of GoogLeNet, traditional ShuffleNet, VGGNet, ResNet and improved ShuffleNet through tire database verification. The experiment found that the detection rate of tire debris defects was 94.7%. Tire defects can be effectively detected, which proves the robustness and effectiveness of the improved ShuffleNet, enabling drivers and tire manufacturers to save labor costs and greatly reduce tire defect detection time.

## Introduction

Tire failure is the last thing a driver wants to encounter when driving a car at high speed, but it is also the most difficult to prevent. As one of the most important parts of a vehicle, defects such as small pits and slight cracks on the tire surface may cause major traffic accidents and directly affect the driving safety of the vehicle. Therefore, tire quality guarantees the normal driving of the car, tires are very important but few drivers regularly monitor their condition. Drivers are aware of the risks of improper inflation, but continue to use vehicles with underinflated tires. And for lesser-known problems like tire aging, oxidation and cracking, drivers don't realize the risks and give up inspections. Using an easy-to-use visual tire inspection system will increase driver awareness and increase adherence to proper tire change times, thereby reducing the risk of sudden separation of tire material or a puncture. In recent years, machine vision detection technology has developed rapidly, and its high efficiency, stability and high degree of automation have laid a theoretical foundation for the development of tire defect detection systems.

Based on image processing technology, images obtained by various imaging methods are analyzed, processed and processed. To make it meet visual, psychological and other requirements, machine vision detection technology replaces the human eye to make judgments. It is widely used in industrial applications due to its non-contact, wide visual range, high reliability and low cost. According to the type of defect detection algorithm, tire defect detection technology can be divided into traditional visual detection technology^1,2^ and detection technology based on deep learning^3,4^. Traditional visual inspection techniques can be divided into statistical-based methods^5,6^, frequency-domain analysis-based methods^7,8^, and model-based methods^9–13^. The methods of applying frequency domain include Fourier transform, Gabor filter, wavelet transform and Hough transform. Methods of using the model include Markov random field model, Weibull model, total variation model, etc. Behroozinia et al.^7^ analyzed both undamaged and damaged tires using a time-domain and frequency-domain analysis-based method that provided information on the location and length of tire crack defects.

Zheng et al.^13^ proposed tire defect classification using a deep convolutional sparse coding network, and constructed a novel deep convolutional sparse coding network (DCScNet) for tire defect classification. The experimental results verified its excellent classification performance. Among them, statistics-based methods include threshold segmentation^14,15^, clustering statistics^16–18^, edge detection^19,20^, gray value analysis^21–23^, gray co-occurrence matrix^24–26^, local binarization^27,28^, and morphological transformation^29–32^.

Wang et al.^33^ developed a fully convolutional network (FCN)-based defect detection method for tire X-ray images. Experimental results show that the method can accurately locate and segment defects in tire images. Rajeswari et al.^34^ developed a weighted mass-based convolutional neural network to detect tire defects, and the results of this study showed that tire deformation and durability can be predicted from track design through a weighted mass-based convolutional neural network. Yang et al.^35^ developed an improved Faster RCNN-FPN for tire speckle interference bubble defect detection. Their research can fuse features across levels and directions to improve small object detection and localization and improve object detection accuracy. Unsupervised learning and deep learning for tire defect detection analysis have been studied by Kuric et al.^36^. Anomaly detection based on data from laser sensors and using an unsupervised clustering method, followed by a VGG-16 neural network to classify defects.

Deep learning has made great strides in the field of computer vision and its industrial applications in recent years. Compared with traditional visual detection techniques, deep learning-based methods have attracted much attention due to their powerful feature extraction ability and parameter self-learning ability. There are other detection methods based on deep learning^36–41^. LeCun^42,43^ systematically summarized the principles of end-to-end training and determined the structure of modern CNN networks. With the development of high-performance GPUs in computers, Krizhevsky^44^ proposed the AlexNet network framework, ushering in a new era of deep learning. Since then, various excellent network frameworks, such as GoogLenet^45^, ResNet^46^, VGGNet^47^, Squeezenet^48^, ShuffleNet^49^, etc., have been proposed one after another, and the network has gradually developed towards a deeper and lighter weight.

This paper investigates such a machine vision implementation of deep learning, using pictures of vehicle tires that can identify the "normal" or "abnormal" state of the tires to classify the presence of surface cracks. This study proposes a tire defect detection system based on deep learning, which cuts each preprocessed tire image with a fixed pixel size. The segmented images are marked with features by the visual image annotator, and the improved ShuffleNet network is adaptively trained with the prepared training set and test set. Finally, the trained model is used for tire defect detection. Compared with the traditional tire deep learning defect detection performance, the three methods of GoogLeNet, traditional ShuffleNet and improved ShuffleNet are compared. The improved ShuffleNet has higher detection accuracy, can be better applied to model training and testing, and can classify and detect multiple tire defects at the same time. The tire detection model in this paper has been verified by experiments and can effectively detect tire defects, which proves its robustness and effectiveness.

It is believed that the model proposed in this research has significant advantages in the following aspects:

1. Objectivity: Compared to manual experience-based judgments, the model in this research can provide more objective and accurate detection results, thereby reducing the risk of misjudgment.
2. Consistency: The deep learning-based detection method can ensure consistent evaluation of different tires, while manual judgments may be affected by individual experience and skill differences.
3. Automation: The model in this research can automatically identify tire cracks, saving car owners time and effort in inspecting tires, and reducing safety risks caused by negligence.
4. Preventive maintenance: The deep learning-based detection method can identify potential problems before tire cracks pose a danger to the vehicle, helping car owners to perform preventive maintenance in advance.
5. Application scope: Although ordinary car owners may have only four tires, in commercial and industrial applications, such as fleets, logistics companies, and public transport operators, there are many tires. The method proposed in this research has enormous potential value in saving manpower and time resources.

Based on the above advantages, it is believed that the tire crack detection method based on image deep learning proposed in this research is of practical value. In future research, further optimization of model performance and the application to a broader range of scenarios will be explored.

## Research theory, data and methodology

### ShuffleNet network

**Figure 1:**
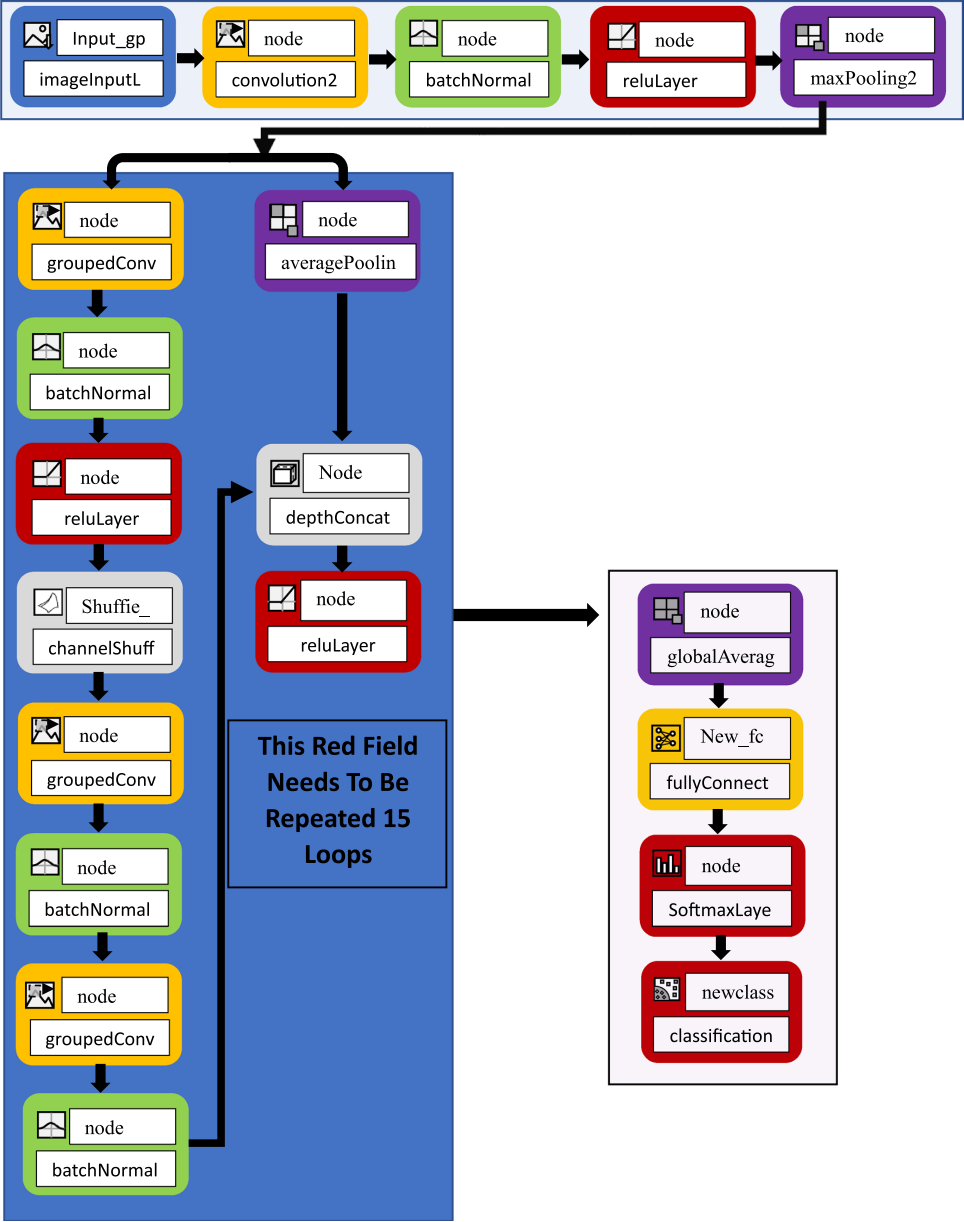
The flow chart of the traditional ShuffleNet neural network^49^.

Researchers released a high-performance lightweight convolutional neural network ShuffleNet network^49^. The author proposes to use point group convolution to improve the operation efficiency of convolution, and the proposed Channel Shuffle operation can realize information exchange between different channels, which helps to encode more information. Compared with many other advanced network models, ShuffleNet greatly reduces the computational cost and achieves excellent performance while ensuring computational accuracy. In fact, grouped convolution was used in the AlexNet network model as early as possible, and some efficient neural network models such as Xception^50^ and MobileNetv2^51^ proposed later introduced depthwise separable convolution on the basis of grouped convolution. Although the ability of the model and the amount of computation can be coordinated, the computation of point-by-point convolution in the model occupies a large part, so the pixel-level group convolution is introduced into the ShuffleNet structure to reduce the 1 × 1 convolution operation. However, the convolution operation can be restricted to each group-by-group convolution, reducing the computational complexity of the model. However, when multiple group convolutions are stacked, the feature information of the output channel will only come from a small part of the input channel where it is located. The output in a group is only related to the input in the group, and the information of other groups cannot be obtained. The information between the groups is not interoperable, which hinders the flow of information between the channels in the group. The input and output channels of ShuffleNet are set to the same number to minimize memory consumption. Assuming that the size of the feature map is $h\times w$, the number of input and output channels are ${C}_{1}$ and ${C}_{2}$ respectively, and the convolution of width and height is 1×1 as an example. Figure 1 shows the flow chart of the traditional ShuffleNet neural network. According to Float Operations (FLOPs) and multiply-accumulate operations (MAC) calculation formula:

1 $$B=h\cdot w\left({C}_{1}\times \left(1\times 1\right)\times {C}_{2}\right)=h\cdot w\cdot {C}_{1}\cdot {C}_{2},$$

2 $$ \begin{aligned} MAC & = h \cdot w \cdot C_{1} + h \cdot w \cdot C_{2} + \left( {1 \times 1} \right) \times C_{1} \times C_{2} \\ & = h \cdot w \cdot (C_{1} + C_{2} ) + C_{1} \times C_{2} \\ \end{aligned} $$

by means of inequality

3 $${(C}_{1}+{C}_{2})\ge 2\sqrt{{C}_{1}{C}_{2},}$$

thereby

4 $$MAC\ge 2\sqrt{h\cdot w\cdot B}+\frac{B}{h\cdot w}.$$

In the formula: B is FLOPs, $w$ and $h$ are the feature map width and height respectively; MAC is the network layer memory access and read and write consumption costs. Therefore, when ${C}_{1}{=C}_{2}$, that is, when the input channel is equal to the output channel, the memory consumption is the smallest.

### Improved ShuffleNet network

**Figure 2:**
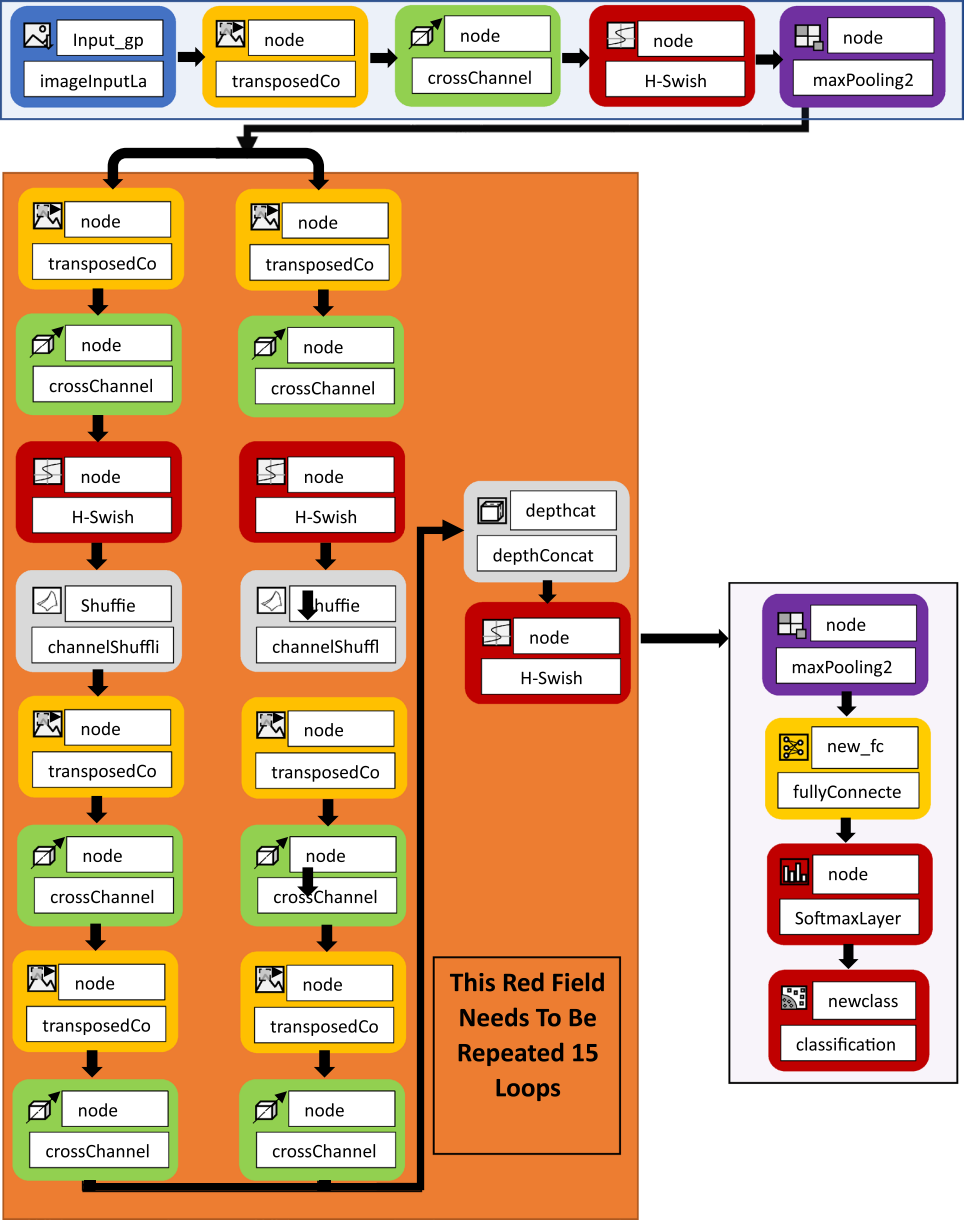
The flowchart of the improved ShuffleNet neural network.

The research proposes the problem of improving the parallel network structure of ShuffleNet and the single channel of traditional ShuffieNet. At the beginning, channel segmentation is used to segment the input feature map in the channel dimension, and the number of channels is divided into two branches. Among them, one branch remains unchanged and does the same mapping, and the other branch continuously performs 8 convolutions to replace the traditional average Pooling. The average Pooling is replaced by 8 transposed Convolution2d Layer, cross Channel Normalization Layer, H-Swish Layerchannel, Shuffl, transposed Convolution2d Layer, cross Channel Normalization Layer, transposed Convolution2d Layer, cross Channel Normalization Layer. The features output by the two branches no longer uses pixel-by-pixel addition operations, but concatenate the channels and output the result. And the channel mixing operation is performed to ensure the information exchange between the two branches. Figure 2 is an improved ShuffleNet space downsampling unit, which adds a transposedConvolution2dLayer Transpose Convolution to the branch. In the transposed convolution, the preset interpolation method is not used, it has learnable parameters, and the optimal upsampling method is obtained by letting the network learn by itself. The cross-Channel Normalization Layer operation normalizes each activation using the local responses in different channels. Normalization across channels usually follows the H-Swish operation. Normalization across channels is also known as local response normalization. A 3 × 3 average pooling operation with stride 2 and channel concatenation instead of pixel-wise addition has the advantage that the channel dimension can be enlarged with a small computational cost. Compared with the traditional ShuffleNet, it is the basic unit of the improved ShuffleNet. On the main branch bottleneck feature map, the point-by-point grouping convolution is used and then the channel mixing operation is performed. Improve the flow of information between different groups, followed by a less computationally intensive depthwise separable convolution. This is followed by a pointwise grouped convolution, and finally the two branch pixels are added together. This improvement can improve the accuracy of image classification recognition.

Activation functions play a crucial role in deep learning, primarily by introducing non-linearity in neural networks. Here, we will introduce five common activation functions: Sigmoid, ReLU6, Swish, H-Sigmoid, and H-Swish. The improved ShuffleNet proposes to use the H-Swish activation function. The traditional ShuffleNet network uses the ReLU activation function, and the formula is shown in Eq. (5). The advantage of the ReLU activation function is that the calculation is simple and efficient, and it can effectively alleviate the gradient disappearance and prevent overfitting. The disadvantage is that one side of the function value is 0, causing the negative gradient to be zeroed, and the neuron may no longer be activated by any data. This phenomenon is called neuronal "necrosis," and the equation states:

5 $$ReLU=\mathrm{max}\left(0,x\right)=\left\{\begin{array}{c}x, x>0\\ 0, x\le 0.\end{array}\right.$$

The Sigmoid function is an S-shaped curve, with its mathematical expression as:

6 $$Sigmoid\left(x\right)=\sigma \left(x\right)=\frac{1}{1+{e}^{-x}}.$$

Its range is between 0 and 1. The Sigmoid function can compress any real number into the range of 0 to 1, making it commonly used in binary classification problems. However, due to its vanishing gradient issue (gradient is close to 0 at both extremes) and non-zero centricity, its application in deep learning is less common nowadays.

ReLU6 is a variant of ReLU (Rectified Linear Unit), with its mathematical expression as:

7 $$ReLU6\left(x\right)= min\left(max\left(x,0\right),6\right).$$

The ReLU6 function limits the input between 0 and 6. This means that the output value for the negative part is 0, while the output for the part greater than 6 is 6. Compared to the traditional ReLU function, ReLU6 has a finite output range, which helps alleviate the exploding gradient problem.

From Eqs. (6) and (7), it can be obtained as follows:

8 $$H\text{-}Sigmoid=\frac{ReLU6\left(x + 3\right)}{6}.$$

H-Sigmoid compresses the input value between 0 and 1 while maintaining non-linearity similar to the Sigmoid function but with higher computational efficiency. However, it may also suffer from the vanishing gradient issue.

Swish is a self-gated activation function proposed by the Google Brain team in 2017, with its mathematical expression as:

9 $$Swish(x) = x \cdot \sigma (x)$$

The Swish function can be seen as a combination of the Sigmoid function and a linear function. Compared to ReLU, the Swish function has smoother gradients and performs better in some deep learning applications.

In summary, these activation functions play important roles in deep learning. The Sigmoid function is mainly used for binary classification problems, but its application in deep learning is less common due to the vanishing gradient problem. ReLU6 is a variant of ReLU with a finite output range, helping to alleviate the exploding gradient problem. The Swish function has smoother gradients and performs better in some deep learning applications. H-Sigmoid and H-Swish are simplified versions of the Sigmoid and Swish functions, respectively, with higher computational efficiency, widely used in lightweight neural networks and edge computing scenarios.

H-Sigmoid is a simplified version of the Sigmoid function, with lower computational cost, and its mathematical expression as:

10 $$H\text{-}Swish\left(x\right)= x \cdot \frac{ReLU6\left(x + 3\right)}{6}=\left\{\begin{array}{c}0,x \le -3\\ x,x \ge +3\\ x \cdot (x + 3)/6,otherwise.\end{array}\right.$$

The Swish activation function, as demonstrated in Eq. (9), outperforms the ReLU activation function and considerably enhances the neural network's accuracy. However, its intricate non-linear computation and derivation make it less compatible with the quantization process, resulting in longer calculation times. As a result, this research employs the H-Swish activation function as a substitute for the ReLU activation function to enhance the ShuffleNet model, as presented in Eq. (10).

### Description of the research data

**Figure 3:**
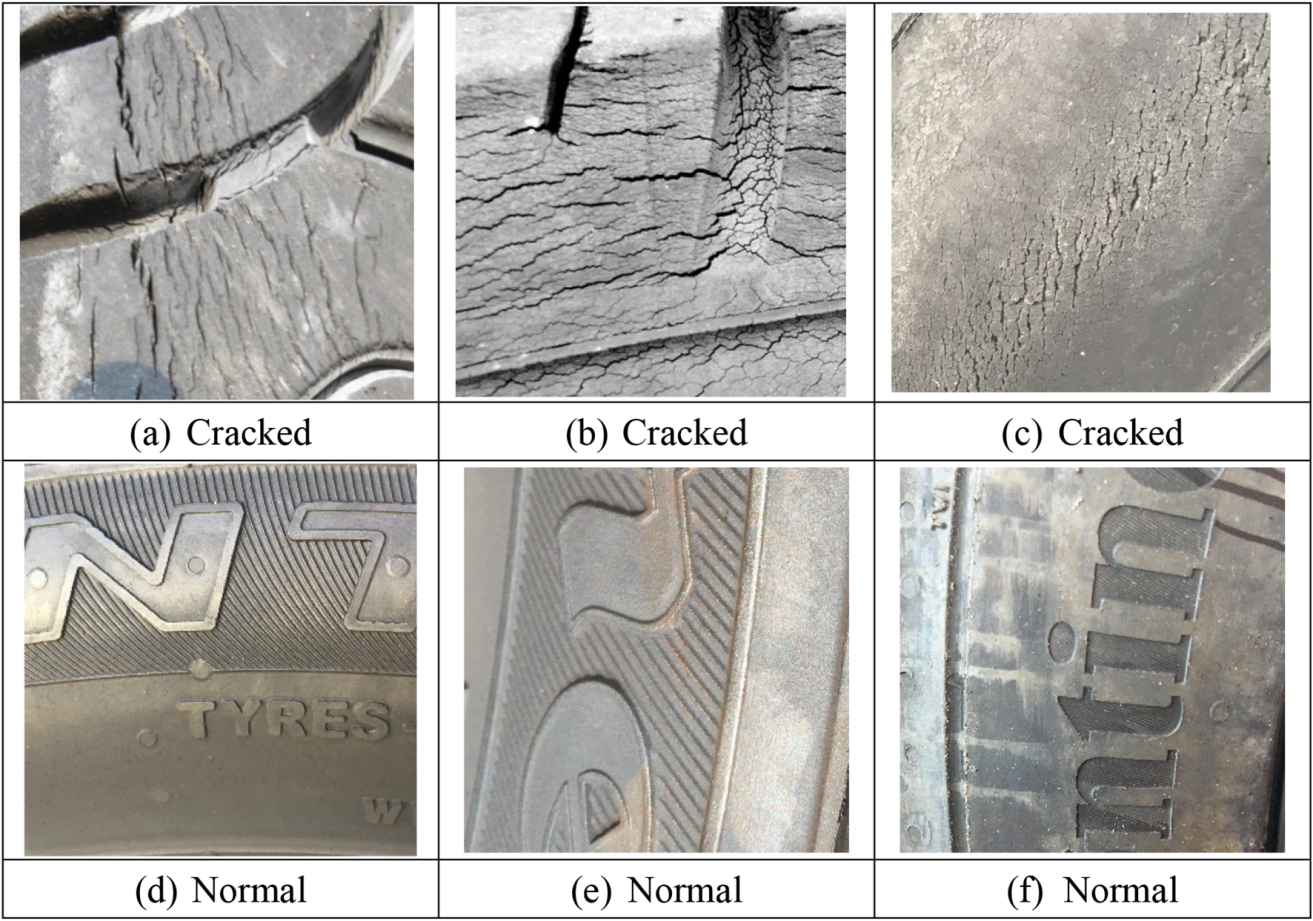
Imagess of "Cracked" and "Normal" tires.

A tire is a flexible composite structure composed of rubber and cord with typical fatigue failure characteristics. The belt cords and carcass cords of all-steel radial tires are made of high-strength steel wires, which are mainly used in medium and heavy-duty trucks that require high bearing capacity. Due to the limitation of the manufacturing process, microscopic defects will inevitably appear inside the tire during the processing and forming process. During the driving process of the vehicle, the alternating load on the tire will cause the expansion of tiny cracks between the belt layers or the end of the carcass turn-up. In addition, the tire will be greatly deformed in the ground contact area due to the alternating load of repeated reciprocation. And the stress and strain of the tire change alternately at a high frequency. This makes the rubber material inside the tire hysteresis, causing the rubber molecular chain to slowly break down. Fatigue failure due to permanent deformation of the tire material structure. At the same time, the hysteresis phenomenon will also cause the heating effect of the rubber material. Part of the energy is converted into heat, which increases the temperature of the rubber material and changes the physical properties of the material. Sidewall and tread images differ between oxidized and non-oxidized (normal) tires, but oxidized tires have a variety of appearances based on compound, age, and exposure to sunlight and chemicals. Some oxidized tires develop long, narrow cracks, while others have a checkerboard appearance. This study validated the database using oxidized and non-oxidized tire sidewall and tread image sources Siegel, Joshua^52^ (Michigan State University). The data is described as "Automotive Diagnostics as a Service: An Artificially Intelligent Mobile Application for Tire Condition Assessment" (https://doi.org/10.1007/978-3-319-94361-9_13). 250 photos of oxidized “Cracked” and 250 photos of “Normal” tires are divided into two parts in this database, a total of 175 photos for 70% training and 75 photos for 30% testing. Figure 3 Shows photos of "Cracked" and "Normal" tires.

## Results and discussion

This study uses three deep learning image classification methods to identify, and the three deep learning image classification methods are GoogLeNet, traditional ShuffleNet and improved ShuffleNet and find the method with the highest recognition rate, because there are only 250 images in each category, in order to retain more The images are tested for validation, 70% of the images of each category are trained and 30% are tested for validation, so each category has 175 images for training and 75 images for validation.

Deep learning technology has achieved excellent results in image recognition and target detection neighborhoods, and the detection and recognition rate of large and complex data is generally higher than that of traditional algorithms. The object detection task can be divided into two key subtasks: object classification and object localization. The object classification task is responsible for judging whether there is an object of interest category in the input image, and outputs a series of labels with scores indicating the possibility of the object of interest category appearing in the input image. The self-learning ability of deep convolutional neural network is used to learn image features and classifiers, and image feature extraction and classifier learning are integrated. And neither professional need to choose which features to extract nor which classifiers to be selected manually. Implementing an end-to-end process for image classification, the superiority of this end-to-end learning-to-classify approach makes us increasingly abandon traditional image classification algorithms with incoherent intermediate steps with manual rules. It works directly on raw images with little preprocessing. It automatically performs layer-by-layer feature learning and layer-by-layer correction of feedback errors, so that the entire process is consistently optimized to an overall objective function. Then, the optimized network is used to abstract representations of classified images layer by layer until high-level semantics are given. Thus, the problem of defect recognition is transformed into the problem of image-to-deep neural network objective function optimization learning and layer-by-layer abstract representation of images. Although all tires have different cracks, trained artificial intelligence and pattern matching can extract some common features. Reference images show various ruptured tires in Fig. 3. Deep neural networks have the potential to develop a kernel capable of matching the characteristics of tires with cracked sidewalls based on repeating patterns and edges. The jagged edges of these crack patterns appear similar to tire tread patterns, although a well-optimized classifier should be able to robustly identify tread patterns from cracked sidewalls. Unlike other traditional machine learning classifiers, deep neural networks improve performance when learning patterns such as textures without specialized feature engineering. This simplifies developing a generic model, as the computer learns filters to detect cracks in various degradation modes.

To fairly test the three methods with the same parameter settings, specify the algorithm to be 'sgdm', which uses a stochastic gradient descent (SGDM) optimizer with momentum. Verbose is 0, Verbose Frequency is 40, Max Epochs is 10, Mini Batch Size is 4, Validation Frequency is 4, Validation Patience is 4, Initial Learn Rate is 0.0001, Learn Rate Schedule is none, Learn Rate Drop Period is 10, and Learn Rate Drop Factor is 0.01. L2Regularization is 0.001, Momentum is 0.9, Gradient Threshold is Inf, Gradient Threshold Method is l2norm, Sequence Length is longest, Sequence Padding Value is 0, and Execution Environment is GPU.

The confusion matrix shows the deep learning classification results, which can verify the performance of different deep learning algorithms. Confusion matrix is explained below. Columns correspond to the results for the true class. Rows correspond to the results of predicted classes, and correctly classified observations are shown in diagonal cells. The observed value of image classification of normal tires and faulty tires during the test is 75 images each. In the results images of faulty tires are called cracked, images of normal tires are called normal, and misclassified predictions are shown in off-diagonal units. Each cell displays the percentage of the total number of forecasts and the number of forecasts for a clearer display of the results. The row at the bottom of the graph shows the percentage of correct and incorrect classifications for all examples of image categories of normal and faulty tires. These metrics are often referred to as recall (or true positive rate) and false negative rate, respectively. The rightmost column of the graph represents the percentage of correct and misclassified predictions for all examples that fall into the categories of images of normal and faulty tires. These metrics are commonly referred to as accuracy (or positive predictive value) and false discovery rate, respectively. The lower right cell of the graph shows the overall deep learning algorithm accuracy.

In deep learning, the loss function and the accuracy rate (Accuracy) are used to determine whether the accuracy is better. loss is the loss value calculated by my preset loss function; accuracy is the model obtained on the dataset based on the given label. evaluation results. The deep learning algorithm calculates the ratio of the number of samples correctly classified by the model to the total number of samples through the accuracy of the model on the training and testing data sets to measure the effect of the model. The goal is to measure the effect of the model. Through the calculation of the loss function, the model parameters are updated, and the goal is to reduce the optimization error, that is, under the joint action of the loss function and the optimization algorithm, to reduce the empirical risk of the model.

The main structure of GoogLeNet consists of an input layer, 5 groups of convolution modules and an output layer, including 22 parameter layers and 5 pooling layers. The input layer is a 224 × 224 × 3 image, the first and second groups of convolution modules include convolutional layers and maximum pooling layers, and the third, fourth and fifth groups of convolution modules are mainly composed of Inception Module structure The output layer consists of an average pooling layer, a dropout layer, and a fully connected layer. The Inception module structure has 4 parallel paths. The first 3 paths use 1 × 1, 3 × 3 and 5 × 5 convolution kernels to extract different image receptive field feature information, and the fourth path uses a 3 × 3 pooling kernel to Pick feature points, prevent overfitting, and add a 1 × 1 convolution. In order to reduce the complexity of the model and reduce the data dimension, 1 × 1 convolution operations are added to the second and third paths.

**Figure 4:**
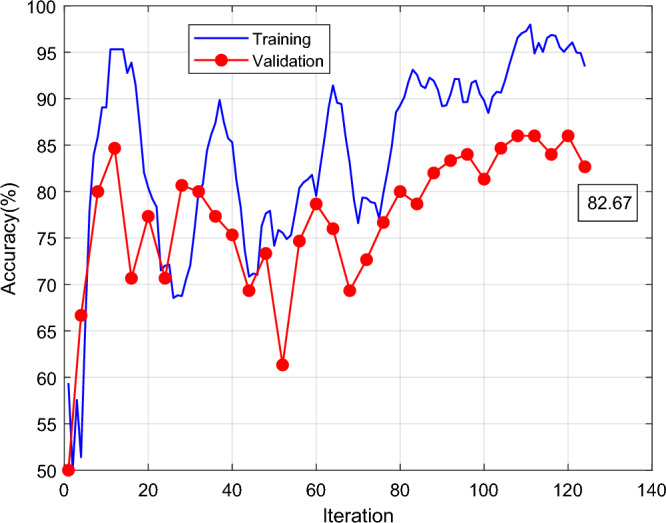
GoogleNet classification accuracy and iterations results.

**Figure 5:**
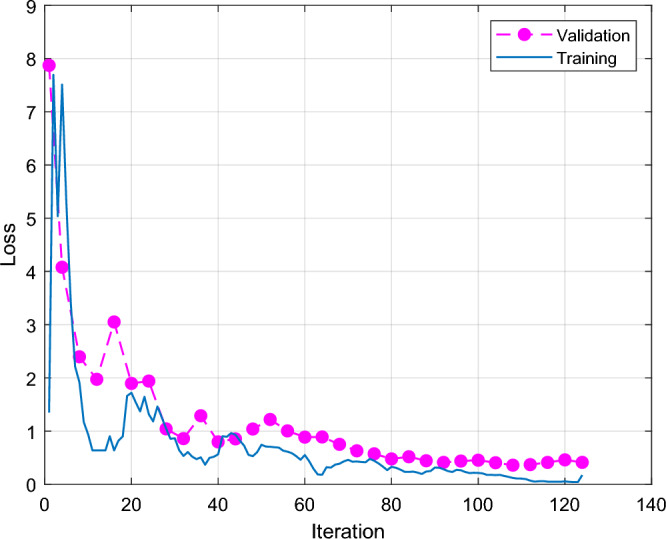
GoogleNet classification loss and iterations results.

**Figure 6:**
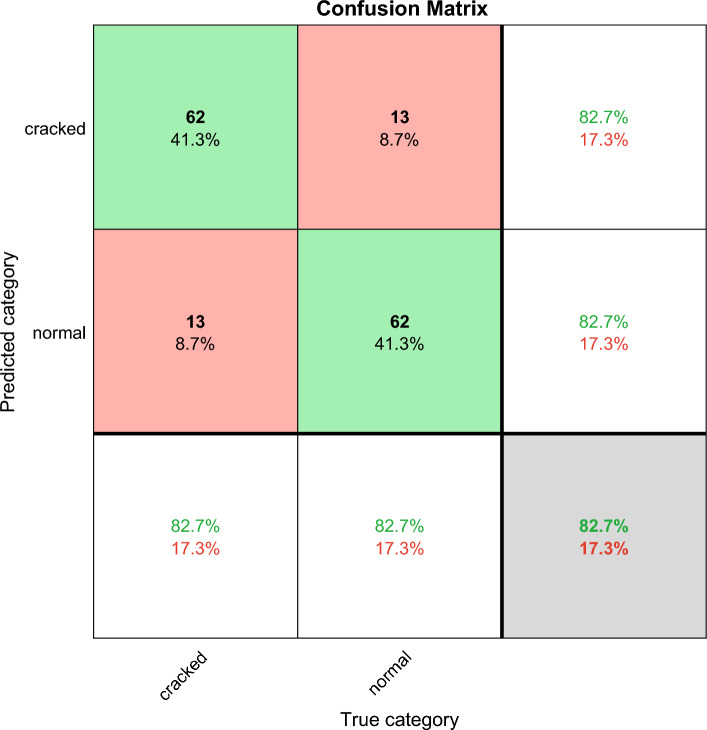
GoogLeNet confusion matrix classification results, the total correct rate is 82.7%.

Figure 4 shows the results of GoogleNet classification accuracy and iterations. The X-axis is the number of iterations, and the Y-axis is the classification accuracy. The blue line is training and the red line is validation. The GoogleNet classification accuracy rises rapidly at the first 10 iterations, training as high as 95% and the red line is the validation as high as 85% at the first 10 iterations. The training and verification cycle is high and low, and the final red line is the verification classification accuracy rate of 82.67. Figure 5 shows the results of GoogleNet classification loss and iterations. Classification results Fig. 6 shows the GoogLeNet classification results, with a correct rate of 82.7%. The classification accuracy rate in both cracked and normal categories is 82.7%, and the prediction is correct 62 correct and 13 incorrect.

**Figure 7:**
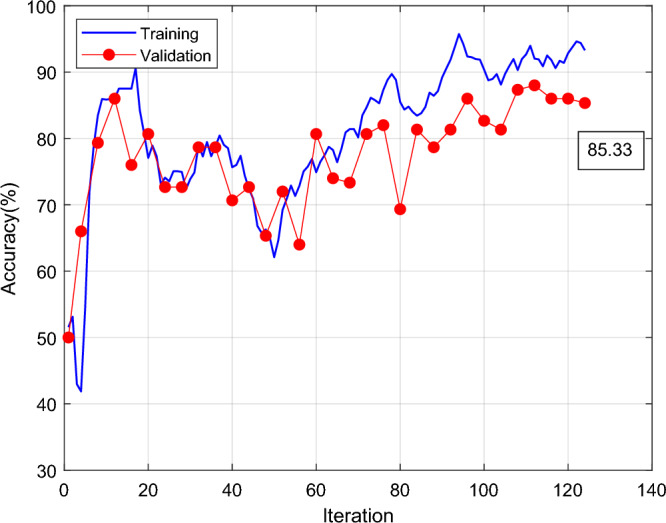
Results of traditional Shufflenet classification accuracy and iterations.

**Figure 8:**
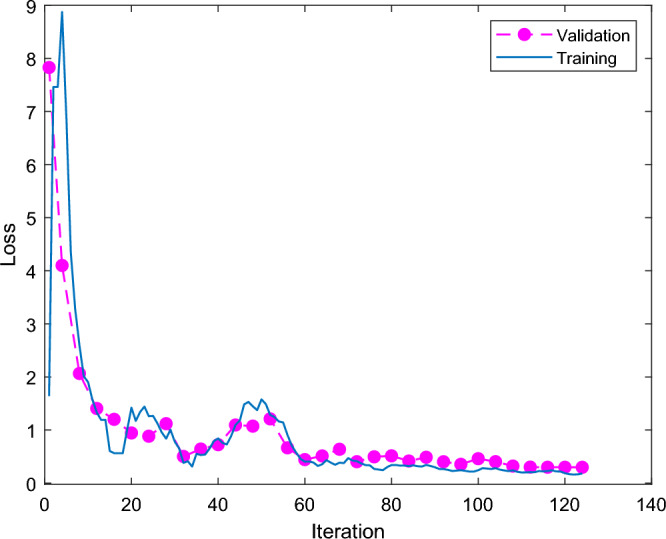
Results of traditional Shufflenet classification loss and iteration times.

**Figure 9:**
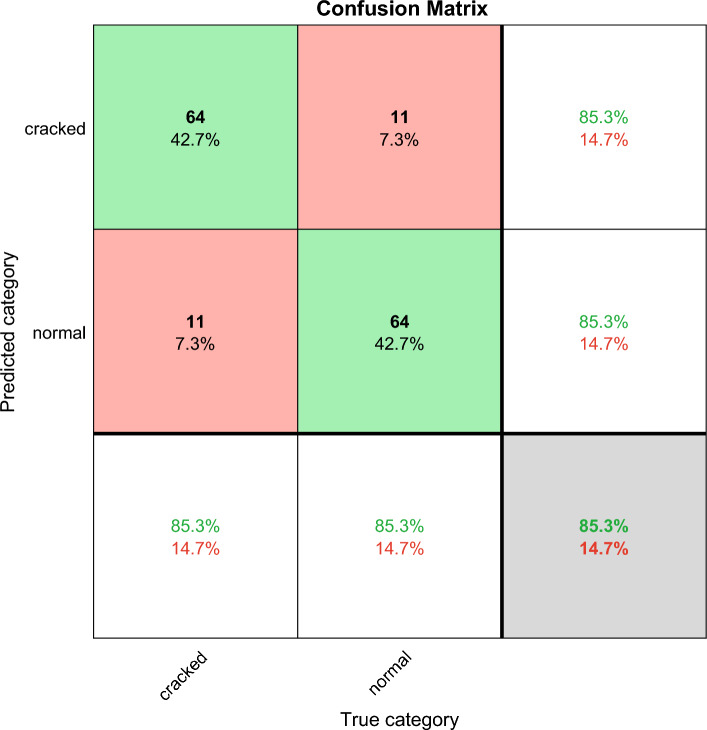
The traditional Shufflenet confusion matrix classification results, the total correct rate is 85.3%.

Figure 7 shows Shufflenet classification accuracy and iterations results. The X-axis is the number of iterations, and the Y-axis is the classification accuracy. The blue line is training and the red line is validation. Shufflenet classification accuracy rises rapidly at the first 18 iterations. The training high reaches 90% and the red line is the validation high reaching 85% at the beginning of 18 iterations. The training and validation curves drop and then slowly rise, and the final red line is the validation classification accuracy of 85.33. Figure 8 shows the results of Shufflenet classification loss and number of iterations. The traditional ShuffleNet classification results are shown in the Fig. 9, and the correct rate is 85.3%. The classification accuracy rate of cracked and normal is 85.3% and the prediction is correct 64 correct and 11 incorrect.

ResNet and VGGNet also have significant features and achievements in the field of image deep learning. The following is an analysis of the characteristics of these two methods in tire crack detection, as well as an analysis of their accuracy results.

**Figure 10:**
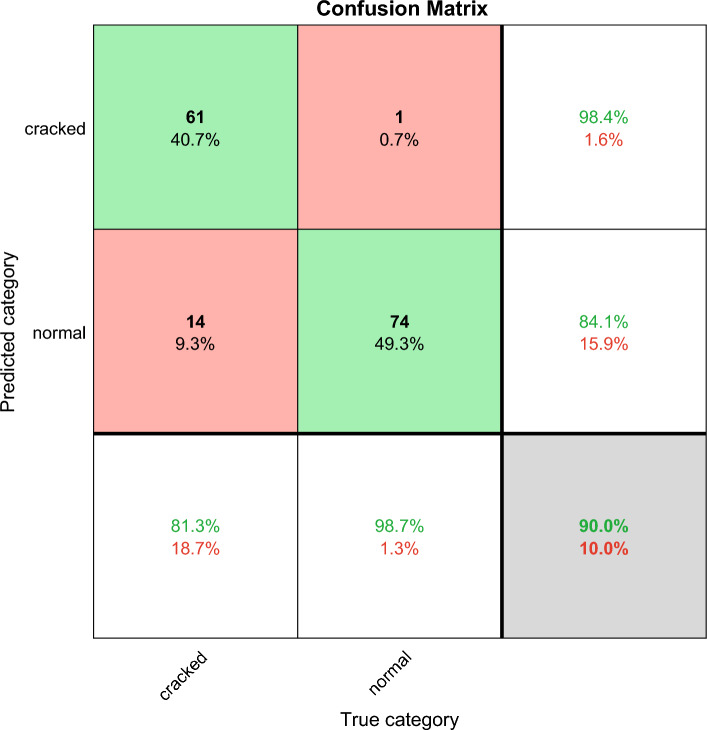
The ResNet confusion matrix classification results, the total correct rate is 90%.

**Figure 11:**
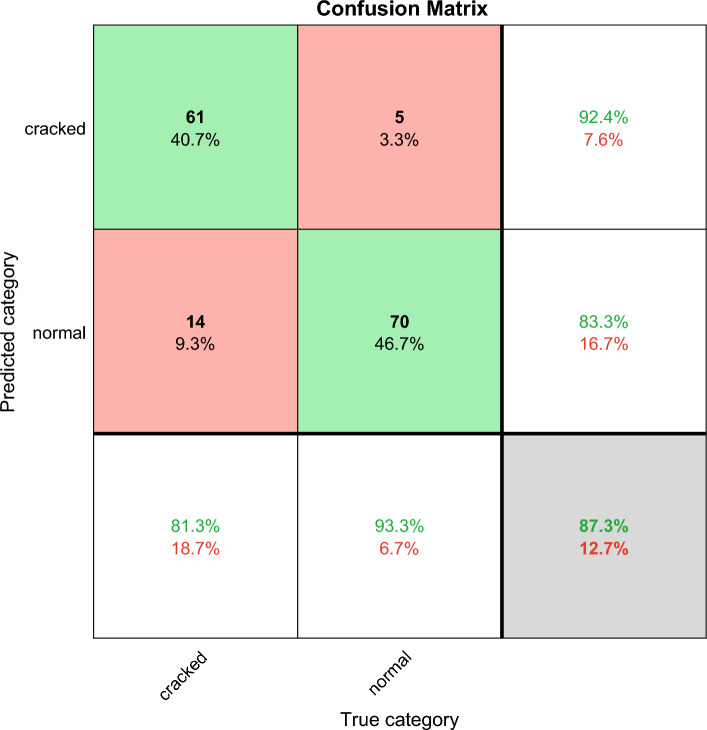
The VGGNet confusion matrix classification results, the total correct rate is 87.3.

1. ResNet (Deep Residual Network): ResNet introduces residual structures to address the vanishing gradient and exploding gradient problems in deep neural networks. In tire crack detection, ResNet can effectively capture the detailed features of the tire, especially the classification of cracks. The accuracy of ResNet in tire crack detection is likely to be around 90% as shown in Fig. 10.
2. VGGNet: VGGNet reduces the number of network parameters and increases training speed by using a stacked structure of multiple 3 × 3 convolutional layers. In tire crack detection, VGGNet can effectively extract local features of the tire and accurately classify cracks. The accuracy of VGGNet in tire crack detection is approximately 87.3% as shown in Fig. 11.

Taking all factors into consideration, although ResNet and VGGNet have potential in tire crack detection.

**Figure 12:**
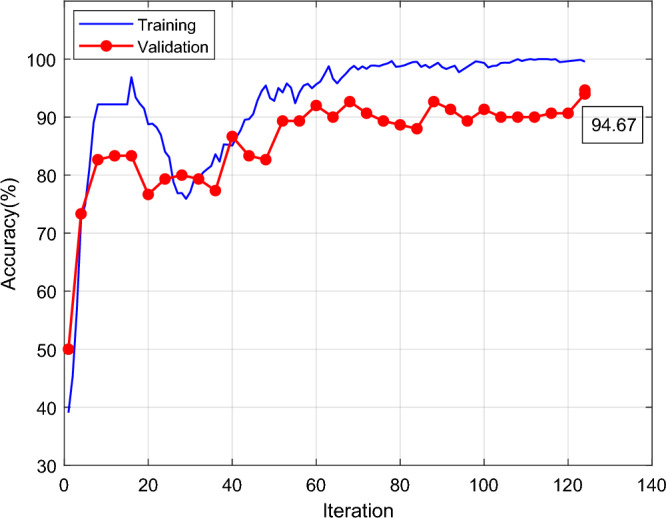
Improved Shufflenet classification accuracy and iterations results.

**Figure 13:**
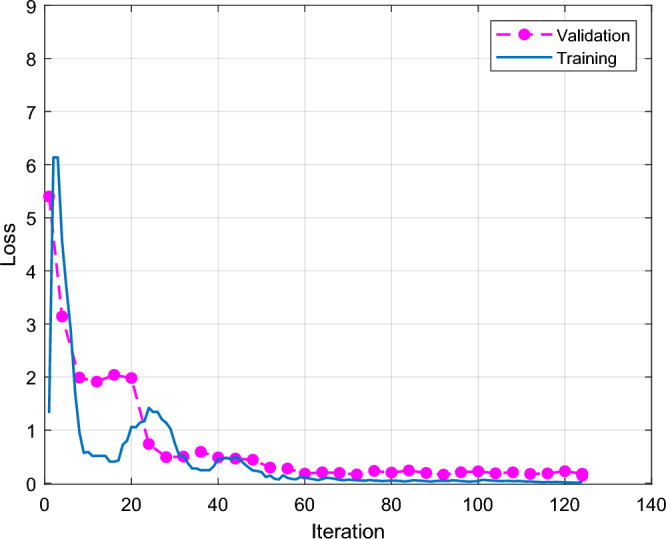
Improved Shufflenet classification loss and iterations results.

**Figure 14:**
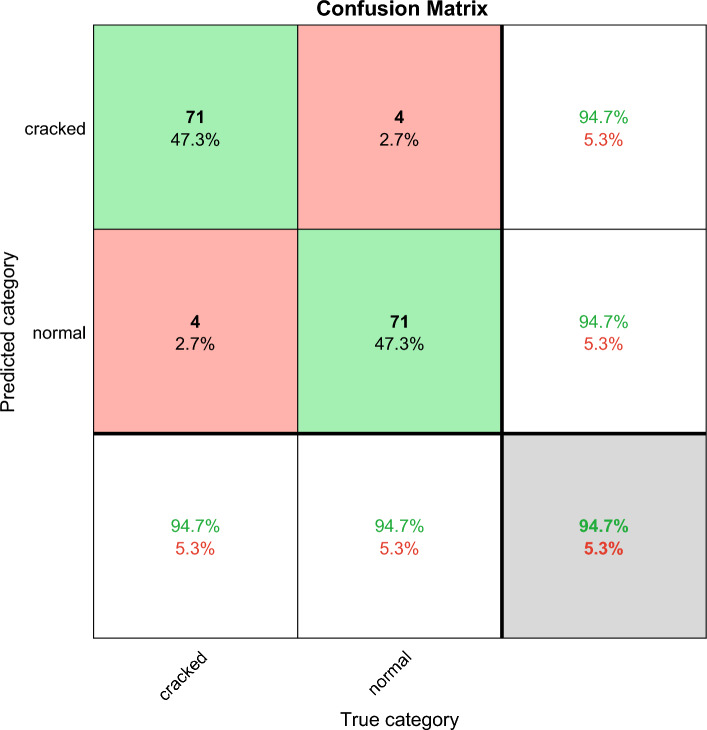
Improved Shufflenet confusion matrix classification results, with a total accuracy of 94.7%.

Figure 12 shows the improved Shufflenet classification accuracy and iterations results. The X axis is the number of iterations, the Y axis is the classification accuracy, the blue line is the training and the red line is the validation. The classification accuracy of the modified Shufflenet increases rapidly at the first 18 iterations. The training height reaches 95% and the red line is the validation height which reaches 84%. At the beginning of 18 iterations, the training and validation curves gradually increase. The improved Shufflenet is close to full score after 70 iterations. The final red line is that the validation classification accuracy is 94.67. Figure 13 shows the results of the improved Shufflenet classification loss and the number of iterations. The improved ShuffleNet classification results are shown in the Fig. 14, the correct rate is 94.7%. Figure 14 shows the confusion matrix of the classification results for the improved ShuffleNet, with an overall accuracy of 94.7%. There are 75 samples for both normal and cracked tire categories. The classification accuracy for the normal category is 94.7%, with 71 samples correctly predicted as normal tires and 4 samples mistakenly predicted as cracked tires. Similarly, the classification accuracy for the cracked category is 94.7%, with 71 samples correctly predicted as cracked tires and 4 samples mistakenly predicted as normal tires.

In this study, the YOLOv7 method was employed to detect tire crack locations, aiming to enhance the identification capability for tire cracks. A collection of tire images with various sizes of cracks was used for training and testing to evaluate the effectiveness of YOLOv7 in tire crack detection.

**Figure 15:**
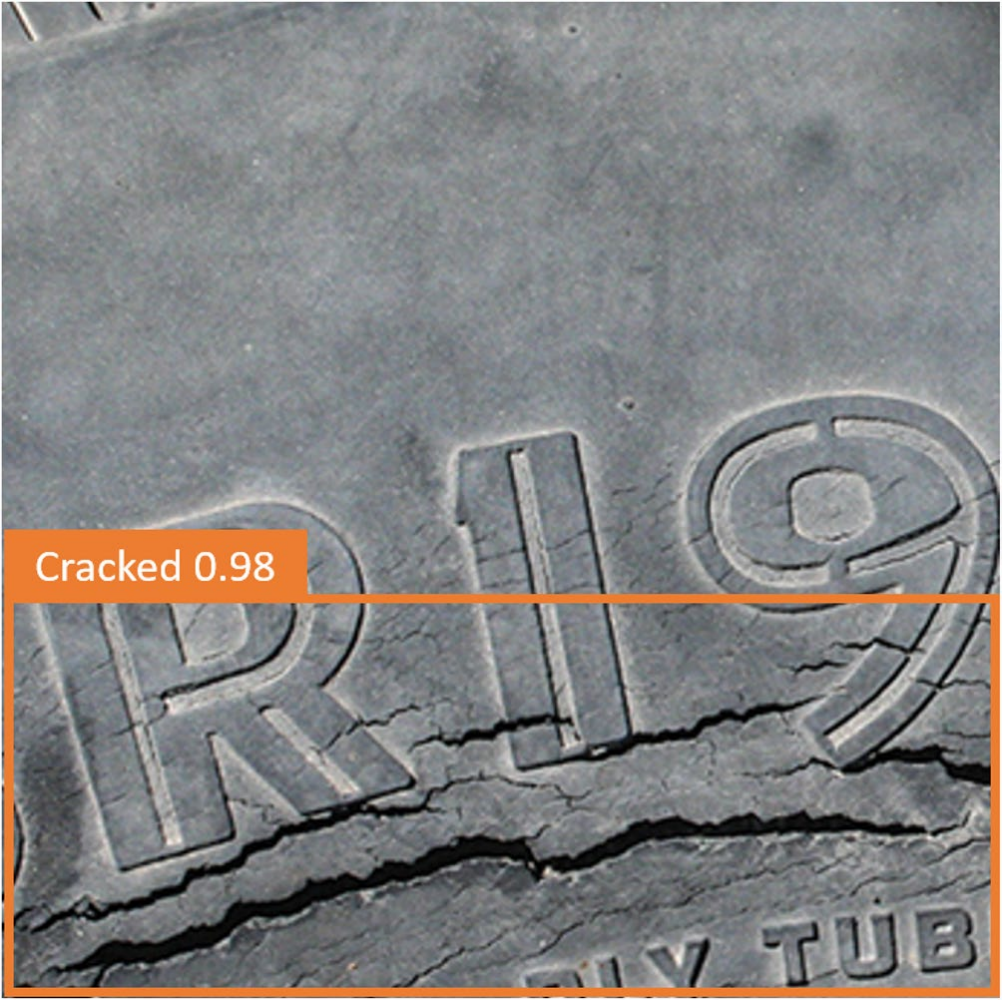
In YOLOv7, the detection results of large cracks in defective tires are 98%.

**Figure 16:**
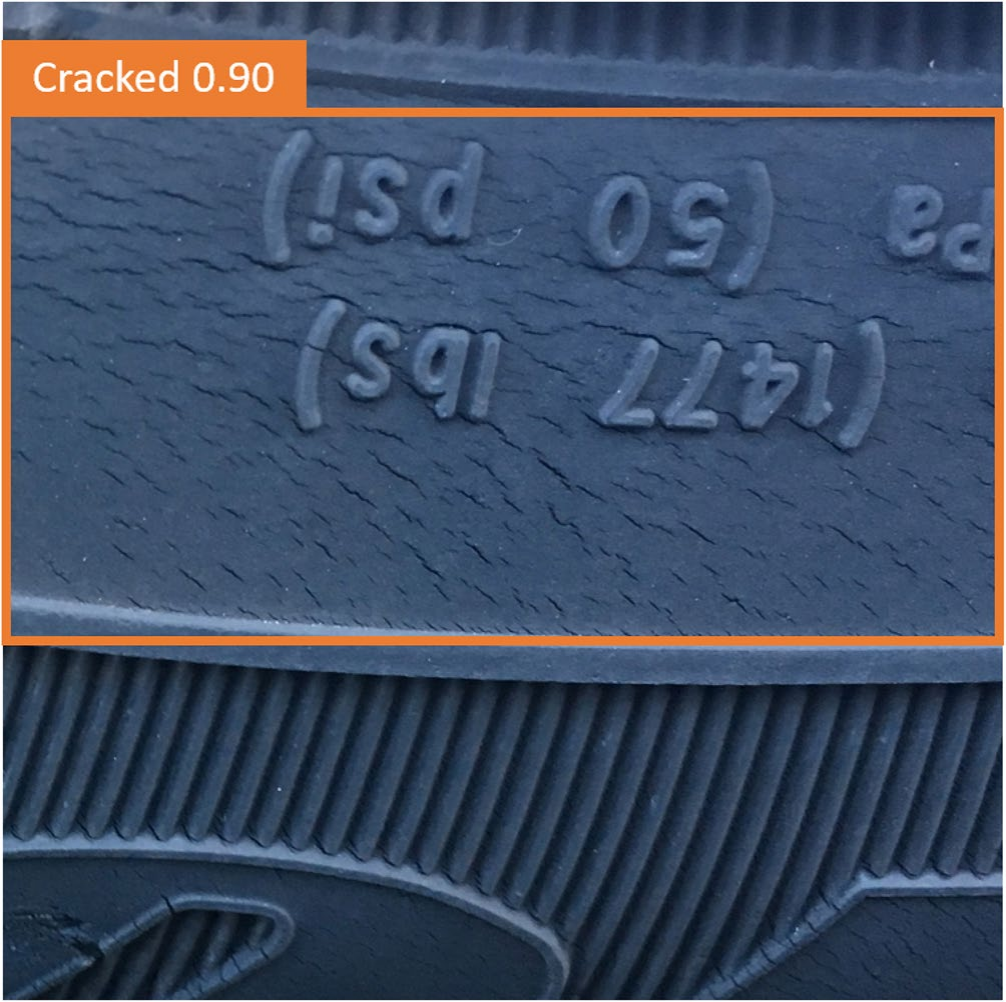
In YOLOv7, the detection results of small cracks in defective tires are 90%.

Upon training and testing the dataset, significant results were achieved using YOLOv7 in detecting tire crack locations. The method was able not only to accurately identify tires with cracks but also to determine the specific location of the cracks. In the test dataset, YOLOv7's accuracy rate reached 92.3%, demonstrating its effectiveness in tire crack detection. Additionally, this study compared the detection performance of YOLOv7 on cracks of different sizes. The results showed that the method excelled in detecting larger cracks, with an accuracy rate as high as 98%, as illustrated in Fig. 15. For smaller cracks, YOLOv7 still exhibited good identification capabilities, with an accuracy rate of 90%, as depicted in Fig. 14. This indicates that YOLOv7 has strong robustness in detecting cracks of various sizes (Fig. 16).

In summary, this study believes that YOLOv7 demonstrates superior performance in detecting tire crack locations. This study provides an effective technical means for tire crack detection and offers strong support for further optimization of algorithms and expansion of application scope in the future.

Table 1 shows all methods compared in accuracy. In this study, GoogleNet, Traditional Shufflenet, VGGNet, ResNet and Improved Shufflenet achieved accuracies of 82.7%, 85.3%, 87.3%, 90% and 94.7%, respectively, demonstrating good performance in image classification and object detection. Traditional method may not be able to achieve higher accuracies compared to the Improved Shufflenet in this study. Moreover, due to limitations in computational resources and training time, this study chose to focus on improving Shufflenet to achieve more efficient and accurate tire crack detection. In future research, this study will investigate how to combine the advantages of traditional method to enhance the accuracy and efficiency of tire crack detection. This may involve exploring shallower network structures to reduce computational resource requirements while maintaining tire crack detection capabilities. Additionally, this study will seek to leverage multimodal data fusion, such as combining optical and thermal imaging data, to improve the reliability of crack detection. This will help further enhance the performance of tire crack detection algorithms in practical applications and broaden their scope of application.

**Table 1:** All methods compared in accuracy.

	Total accuracy (%)
GoogleNet	82.7
Traditional Shufflenet	85.3
VGGNet	87.3
ResNet	90
Improved Shufflenet	94.7

In order to properly use the visual car tire inspection system, the following are suggested:

1. System installation: Collaborate with professional technicians to install the system at suitable locations, such as service centers, tire shops, or parking lots, to provide easy access for vehicle owners.
2. User-friendly interface: Design an intuitive user interface that allows users to easily navigate through the system and access tire inspection results.
3. Detailed instructions: Provide clear instructions on the proper placement of the vehicle and tire alignment to ensure accurate results.
4. Real-time feedback: Offer real-time feedback on the tire inspection results, enabling users to make informed decisions on tire maintenance or replacement.
5. Automatic updates: Implement automatic updates to the system to ensure it stays up-to-date with the latest tire inspection algorithms and techniques.
6. Customization: Allow users to customize settings, such as the level of detail in the inspection results, according to their needs.
7. Integration with maintenance services: Integrate the visual car tire inspection system with maintenance services, so users can easily schedule appointments for tire maintenance or replacement based on the inspection results.
8. Data storage and analysis: Store tire inspection data securely and enable users to analyze historical data to track the performance and condition of their tires over time.

By implementing the above recommendations, the installation and usage of the visual car tire inspection system will become easier, and it will play a more significant role in practical applications. In further research, the system's performance can be continuously optimized, and its application scope can be expanded to cover more aspects of tire inspection, such as tire wear, pressure, and balance.

## Conclusion

This paper proposes a high-precision tire defect detection system. Through the trained and improved deep learning ShuffleNet model, the tire images are detected for defects. Through the tire database experiment, it can be proved that the algorithm in this paper can accurately detect tire crack defects. In the research results, the classification accuracy of GoogLeNet is 82.7%, the traditional ShuffleNet is 85.3%, VGGNetis 87.3%, ResNet is 90%, and the improved ShuffleNet is 94.7%. This research method can be used not only for driving but also for tire factories. The improvement of traditional tire surface defects relies on manual detection, which has the problems of high labor intensity, large subjective influence by people and low efficiency. It can meet the tire manufacturer's problem of tire debris defect detection, and achieve the effect of classifying and detecting defects at the same time. Several study limitations here are work for future research. This paper focuses on the study of normal tires and tire oxidation crack defects, so the detection method of this study has limitations. However, actual tire defects are diverse and complex, including air bubbles, foreign objects, cracks, and other types. In this study the validity of the detection algorithm is not discussed to detect crack specifications, it can be discussed in the future.

## Data Availability

The datasets and the code used and/or analysed during the current study are available from the corresponding author on reasonable request.
